# Unusual Sites of Cutaneous Tuberculosis: A Report of Two Cases

**DOI:** 10.1155/2017/7285169

**Published:** 2017-02-27

**Authors:** Dimple Chopra, Vishal Chopra, Aastha Sharma, Siddharth Chopra, Shivali Aggarwal, Deepak Goyal

**Affiliations:** ^1^Department of Dermatology, Government Medical College, Patiala, Punjab, India; ^2^Department of Pulmonary Medicine, Government Medical College, Patiala, Punjab, India; ^3^Government Medical College, Patiala, Punjab, India

## Abstract

Cutaneous tuberculosis (CTB) is an uncommon small subset of extrapulmonary tuberculosis, comprising 1–1.5% of all extrapulmonary tuberculosis manifestations, which manifests only in 8.4–13.7% of all tuberculosis cases. Lupus vulgaris (LV) and tuberculosis verrucosa cutis (TBVC) are forms of reinfection tuberculosis and often occur in presensitized patients, by exogenous inoculation. We report two cases of cutaneous tuberculosis at unusual sites. A 35-year-old female having a forehead lesion for 2 years was diagnosed as having tuberculosis verrucosa cutis and another 16-year-old girl with lesion in left axilla for 10 years was proven to have lupus vulgaris. The delayed diagnosis was possibly due to lower clinical suspicion due to the presentation of CTB at unusual sites. This highlights the importance of keeping TB as an important differential as misdiagnosis or delayed diagnosis of this entity can lead to prolonged morbidity.

## 1. Introduction

Cutaneous tuberculosis (CTB) comprises a small fraction (2%) of incident cases of TB [1] and the incidence has decreased from 2% to 0.5% [2]. It can present in many different manifestations. The potentiality of skin to react in many different ways to a single agent is nowhere better illustrated than in tuberculosis [3]. The site and clinical picture of CTB can at times be confusing leading to a delay in diagnosis of the disease as happened in these two cases. We report a case of tuberculosis verrucosa cutis (TBVC) presenting as a warty lesion on the forehead and of lupus vulgaris (LV) in the axilla which is a rare presentation.

## 2. Case Report

### 2.1. Case  1

**Figure 1:**
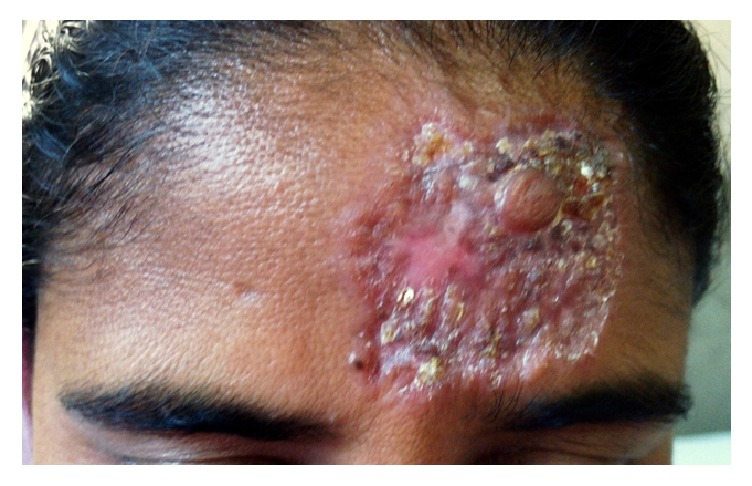
Plaque with warty surface on forehead.

35-year-old female presented with a hyperkeratotic plaque on the left side of forehead. Lesion started as a small papule after 3 months following a roadside fall 2 years back, gradually increased in size, and spread in an annular fashion to form a plaque with warty surface [Figure 1], which started showing ulceration in last 3 months. Lupus vulgaris and leprosy were kept as the differential diagnosis. A detailed history and clinical examination were not in favour of leprosy. She had no history of visit to/residence in known endemic areas for leprosy, no family history of leprosy, no hypo/anaesthesia, tingling, or numbness of hands or feet, and no reduced sweating, loss of hair, dryness of eyes, or epistaxis. On clinical examination an erythematous annular hyperkeratotic plaque with a warty surface measuring 4 × 5 cm was present on the left side of forehead. The lesion showed some crusting and a small scar at the periphery. There was no lymphadenopathy. On clinical examination there were no hypopigmented lesions on body, there was no loss of sensations, and there was no nerve enlargement. Systemic examination also did not reveal any abnormalities. Routine investigations and X-ray chest were normal. Tuberculin skin test was positive with an induration of 20 mm.

**Figure 2:**
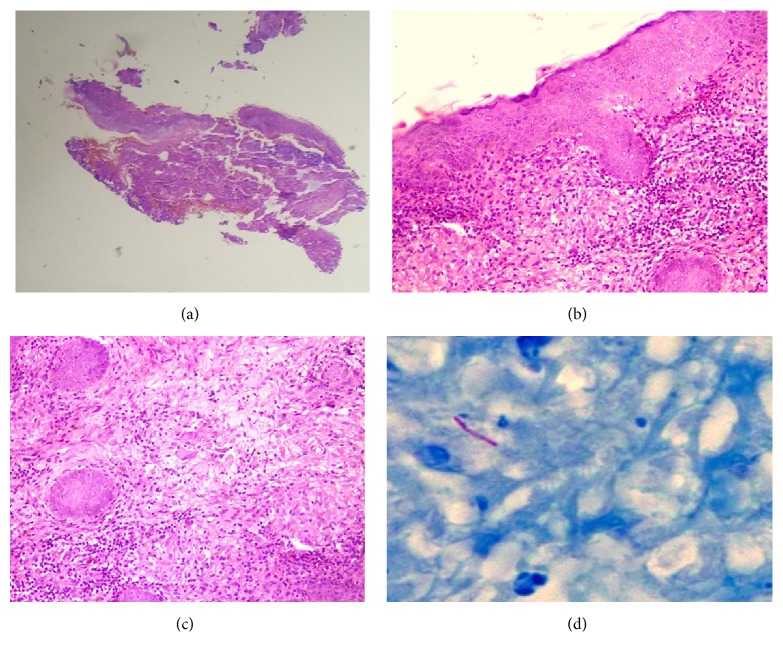
(a) Pseudoepitheliomatous hyperplasia with hyperkeratosis and acanthosis [H and E (40x)]. (b) Pseudoepitheliomatous hyperplasia with hyperkeratosis and acanthosis [H and E (400x)]. (c) Dense inflammatory infiltrates with epithelioid cells and giant cells in the dermis [H and E (400x)]. (d) Ziehl-Neelsen Staining showing scant AFB [ZN Staining (1000x)].

A punch biopsy from the lesion showed pseudoepitheliomatous hyperplasia with hyperkeratosis, acanthosis [Figure 2(a), H and E (40x), and Figure 2(b), H and E (400x)], and dense inflammatory infiltrates with epithelioid cells and giant cells in the dermis with few acid fast bacilli (AFB) [Figure 2(c), H and E (400x), and Figure 2(d), ZN Staining (1000x)]. PAS staining was not performed.

A small scar was evident in the plaque, possibly secondary to an earlier biopsy. Also the histopathology was more in favour of TBVC than lupus vulgaris. A diagnosis of tuberculosis verrucosa cutis was made, and the patient was started on category 1 treatment as per Revised National TB Control Programme (RNTCP) with which the lesions improved.

### 2.2. Case  2

**Figure 3:**
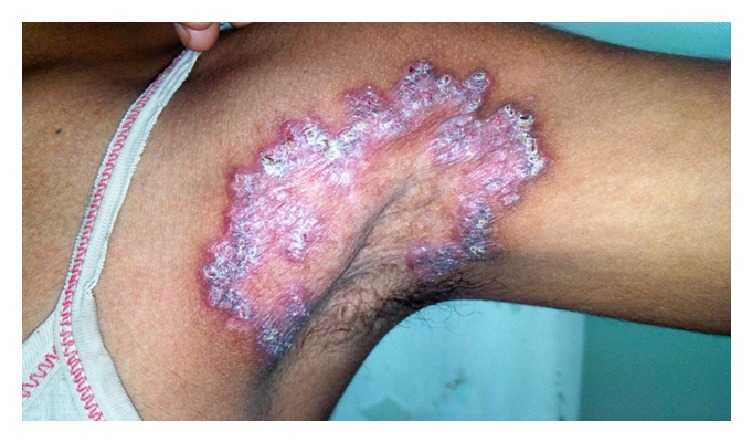
Erythematous scaly annular plaque in left axilla extending up to upper inner arm.

A 16-year-old girl presented to our OPD with a lesion in left axilla [Figure 3] which started 10 years back as a small papule in axilla which turned into a pustule within a week which crusted and spread slowly over years.

Patient did not give any history of trauma, prolonged cough, sputum production, fever, night sweats, and weight loss. No family member had cough or sputum production or history of treatment for tuberculosis.

On examination the lesion was sharply demarcated 10 × 6 cm erythematous scaly annular plaque showing peripheral extension and central clearing with atrophic scarring. The plaque extended up to upper inner arm. On diascopy apple jelly nodules were appreciated. No BCG scar was present. There was no axillary or peripheral lymphadenopathy. Tinea corporis was considered as a differential diagnosis, but the lesion was asymptomatic and other flexures and nails were normal. PAS staining was not performed.

Routine investigations and X-ray chest were normal. Tuberculin skin test was positive with an induration of 18 mm.

**Figure 4:**
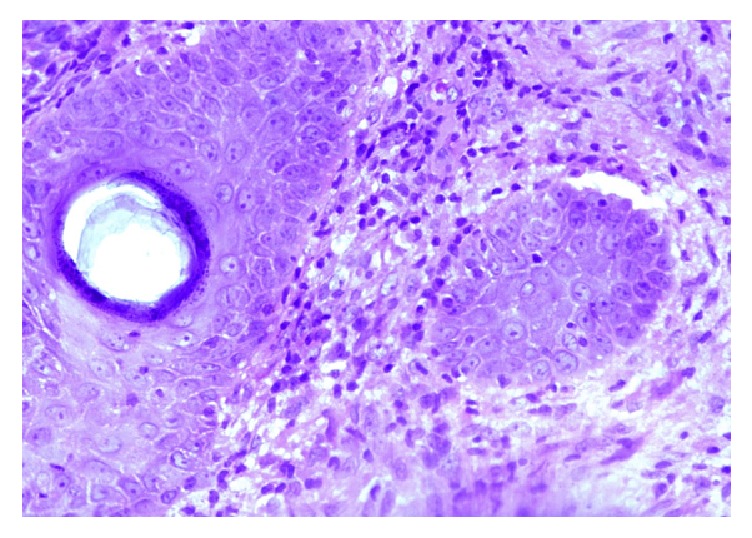
Biopsy shows well-formed granulomas with epithelioid cells and Langhans giant cells surrounded by chronic inflammatory cells and central necrosis [H and E (400x)].

Biopsy revealed well-formed granulomas with epithelioid cells and Langhans giant cells surrounded by chronic inflammatory cells and central necrosis without AFB [Figure 4, H and E (400x)]. PAS staining was not performed.

Diagnosis of lupus vulgaris was made and she was put on category 1 treatment as per Revised National TB Control Programme (RNTCP) with which the lesions improved.

## 3. Discussion

Cutaneous tuberculosis is a rare form of TB and presents with nonspecific and varied clinical presentations. Estimated incidence of this disease is about 0.1% of total patients attending the dermatology outpatient department [4]. Scrofuloderma is the most common form of cutaneous TB in India (50% cases) followed by lupus vulgaris in 42.86%, tuberculosis verrucosa cutis in 4.76%, and lichen scrofulosorum in 2.38% cases [5].

Other than* Mycobacterium TB* the infection can rarely be caused by* Mycobacterium bovis* or other atypical mycobacteria. Pulmonary TB, at present or in the past, is an important risk factor though it was absent in both of the cases. It is more at the sites with trauma as was in the first case though the site was rare. Limbs are the more common sites for cutaneous TB in India [6, 7] whereas neck sites followed by face and trunk are common sites of involvement in the western world.

Various classifications have been proposed to classify cutaneous tuberculosis. Earlier classification was based on the morphology of the lesions. Lai-Cheong et al. later proposed a classification based on the route of infection whether exogenous or endogenous [8, 9]. Now the recent classification based on bacterial load was proposed [10]. It was classified into paucibacillary forms (e.g., lupus vulgaris, tuberculosis verrucosa cutis, and tuberculids) and multibacillary forms (e.g., scrofuloderma, tuberculous chancre, and acute military tuberculosis).

Tuberculosis verrucosa cutis, a paucibacillary form of cutaneous tuberculosis, occurs in those individuals who have already had tuberculosis and have developed a moderate-to-high degree of immunity against these organisms. It is also known as warty tuberculosis, Prosector's wart, butcher's wart, and verrucous tuberculosis [11]. Tuberculosis verrucosa cutis results from inoculation and has an exogenous route. The sites commonly involved are hands in adults and lower extremities in children [12]. The involvement of forehead in this entity is extremely rare. It is usually solitary but multiple lesions may occur. The lesion typically starts with a painless, dusky red, firm, indurated nodule or papule that expands peripherally and is surrounded by inflammation. Spontaneous central resolution with areas of atrophy surrounded by a verrucous keratotic surface or an annular plaque with a warty advancing border is seen. Occasionally pus and keratinous material may be expressed from fissures in the warty areas. Lymphadenopathy is usually absent and, if seen, indicates secondary infection [13].

Histopathology shows pseudoepitheliomatous hyperplasia with marked hyperkeratosis, acanthosis, and dense inflammatory infiltrates with epithelioid cells and giant cells in the mid-dermis. Typical tuberculosis granulomas with characteristic caseation are not common [10]. Mantoux reaction is markedly positive as also in this case. Diagnosis is usually confirmed by typical clinical appearance, histopathological pattern, and a positive response to antitubercular treatment. The differential diagnoses include blastomycosis, chromomycosis, fixed sporotrichosis, lesions caused by nontubercular mycobacteria, lupus vulgaris, and tertiary syphilis.

Lupus vulgaris is the commonest, chronic, progressive, paucibacillary form of secondary cutaneous tuberculosis [12]. It develops in a previously sensitized host having a high degree of tuberculin sensitivity. Lupus vulgaris results both from inoculation and from endogenous spread through hematogenous or lymphatic route from an underlying infective focus [14]. Rarely, it may develop following direct inoculation of the bacilli into skin or at the site of Bacille Calmette-Guerin (BCG) vaccination [15].

Clinically it is characterized by soft reddish-brown plaques with apple jelly nodules on diascopy. The lesions pursue a chronic course over several years and grow by peripheral extension and central scarring. The diverse clinical forms of lupus vulgaris include papular, nodular, plaque, ulcerative, vegetative, and tumour-like lesions. In India, trunk, buttocks, and extremities are the predominant sites affected; in the West the lesions favour head and neck. In this case the lesion was present in the axilla without any history of trauma or any lymphadenopathy which delayed the diagnosis of the lesion. Atrophic scarring of lesions and apple jelly colour on diascopy are characteristic. Histopathologically, it is associated with nonnecrotizing granulomas in which acid fast bacilli are usually not found [16].

The treatment of cutaneous tuberculosis is as per RNTCP guidelines in India.

## 4. Conclusion

Awareness of varied clinical presentations especially at rarer sites with a high index of clinical suspicion of CTB, as in these two cases, is the key to early diagnosis and treatment, thus reducing the morbidity.
